# Computational fluid dynamic modeling of pressure and velocity in a water aspirator

**DOI:** 10.1016/j.heliyon.2025.e42289

**Published:** 2025-01-28

**Authors:** Krzysztof Tadyszak, Mariia Rabyk, Jiří Pánek, Martin Hrubý

**Affiliations:** Institute of Macromolecular Chemistry CAS, Heyrovského nám. 2, 162 06 Prague 6, Czech Republic

**Keywords:** Venturi effect, Turbulent flow, Water pump, Finite element method, COMSOL

## Abstract

This study presents the experimental and theoretical modeling results of pressure changes caused by fluid flow in a water aspirator (water pump) whose working principle is based on the Venturi effect. Experimentally measured pressure drop in a glass-made device is modeled in COMSOL Multiphysics by previously reproducing the device geometry. Computations were performed using a Fluid Flow Module with turbulence model RANS *k-ε*. Pressure and liquid velocity magnitude maps were drowned, and selected vertical and perpendicular cross-sections of velocity and pressure fields were shown and discussed, indicating model limitations.

## Highlights

• Reversed engendered CFD pressure and velocity calculations in a glass-type water aspirator are performed.
• Pressure and velocity maps, as well as selected cross-sections indicating model limitations, are shown and discussed.
• Cavitation probability is discussed, and the cavitation number is estimated.
• Maps of shear stress, Reynolds number, and turbulence parameters are shown.

## 1 Introduction

The results presented in this article are addressed to students who start their adventure with fluid dynamics and would like to deepen their knowledge in this field. The concepts of the static, dynamic pressures and velocity fields are presented. The Venturi effect, which lies at the core of the working principle, is valid not only for liquids shown here but also for gases and can be found in many “real-life” devices used today. In general, a fluid is a substance that deforms continuously when subjected to shear stress, no matter how small that shear stress could be [1].

The Venturi effect is the fluid pressure reduction when the fluid's velocity increases. A water aspirator has three main parts: a nozzle, a throat, and a side exit tube (tenon), where underpressure appears due to fluid flow from the nozzle through the throat.

This effect was discovered in the 18th century. It is the outcome of Bernoulli's principle, which states that an increase in the speed of a fluid coincides with a decrease in static pressure or the fluid's potential energy. Bernoulli equation in one of its forms $\frac{1}{2}{\rho v}^{2}+\rho gz+p+p_{loss}=const\text{.}$, where v is the fluid velocity, ρ is the fluid density, p is static pressure, g - is the acceleration due to gravity, z - height (vertical direction, $z_{\max.}-z_{\min.}=z$), $p_{loss}$ – pressure loss due to friction and other reasons [[1], [2]]. This low can be used for incompressible fluids and maintains constant energy in the system. The first part $\frac{1}{2}{\rho v}^{2}=q$ is called velocity head (or dynamic pressure, or kinetic energy), and ρgz is called elevation head (gravitational potential energy) is due to the fluid's weight, the gravitational force acting on a column of fluid, and p pressure head which is static pressure applied to cause fluid flow. The sum of elevation head (ρgz), static pressure (p), dynamic pressure (q), which is ρgz+p+q is called total pressure [3]. To maintain the validity of this equation, if fluid velocity increases, then static pressure p decreases. Other parameters in this equation for incompressible fluid conditions are constant. An additional term $p_{loss}$ is the resistance head (head loss) appearing due to the frictional forces acting against a fluid's motion by the container [3].

Devices based on the Venturi effect measure fluid (liquid or gas) velocity by measuring relative pressure (Venturi meters, tubes, orifice plates). This effect is used commercially in a vast number of equipment, e.g., in flow meters (dated back to 1888yr.) [4] e.g. liquids in pipes [5], airplanes speed, cargo transportation [6], mixing air and flammable gas - gas stoves, Bunsen burners [7], filtration apparatus [8], removing gas from liquids from the molten salt reactor [9], gaseous pollutants removal [10], bladeless fans [11], airbrushes [12], electro spraying [3], atomizers [13], scrubbers [14,15], carburetors [16], temporary fume hoods [17], pressure inverters [18] or even in pneumatically driven artificial hearts [19]. The effect is also applied in chemical laboratories where glass or stainless-steel water-powered vacuum pumps are used (different names are educator, Venturi pump, and jet pump ejector).

Two additional applications can be found due to conditions present in this device. Both of them are focused on cavitation [[20], [21], [22]]. This is the phenomenon in which the static pressure of a liquid reduces locally to below the liquid's vapor pressure, leading to the formation of vapor-filled bubbles in the liquid. The collapse of the bubbles produces a shock wave (implosion) that can damage even metal parts in its proximity (e.g., nano/microbubble generators [21,[23], [24], [25]]). This effect, which is not limited to aspirators, is also used in vortex diodes [[26], [27], [28], [29]] and can be applied to performing chemical reactions under hydrodynamic cavitation conditions [27,[30], [31], [32], [33]]. It can also serve for flow sterilization, which can be used for bacteria removal [34] and wastewater treatment [35].

Molecular dynamic simulations [36] have shown that the same Venturi effect can be found in the nanoscale systems for throats of 0.68 nm and can be used for pumping liquids, mixing, and obtaining nanoparticles for drug delivery, even in the smallest microfluidic devices [37,38].

The article by Gallitto et al. [7] provides additional historical background on the devices exploiting this effect. Laboratory vacuum equipment can be expensive and unavailable at institutions with small budgets; this does not apply to water aspirators, which can be created using 3D printing technology entirely from commercially available polymers [39].

The most significant disadvantages of water-powered aspirators are low-strength vacuum, water-intensive usage, and mixing potentially hazardous chemicals into the water stream. The vacuum achieved is limited by vapor pressure, which for water is 1.2 kPa (at 10^o^C), 1.7 KPa (at 15^o^C), 2.3 kPa (at 20^o^C), and 3.2 kPa (at 25^o^C) [40]. Experimentally, aspirators can reach similar pressure values ca. 1.3 kPa [15], 2.7 kPa [39,41], 60–70 kPa [42]; for steam aspirators, it is 10 kPa [43] if multiple stages are used 0.4 Pa^43^. For comparison, the standard atmospheric pressure is ca. 101.3 kPa.

The article shows a model of the pump's performance using the finite element method (COMSOL Multiphysics - Fluid Flow Module) focusing on the liquid's velocities and pressures for conditions found in chemical laboratories.

## 2 Materials and methods

### 2.1 CFD model

Finite element analysis was performed with COMSOL ver. 5.6.0.401 - Fluid Flow Module with *Turbulent flow k-ε* *model*. This is the most common computational fluid dynamics (CFD) model to simulate turbulent flow conditions. The program utilizes Reynolds-Averaged Navier-Stokes (RANS) equations, a simplified set of equations used to describe the average behavior of fluid flows [[44], [45], [46]]. The turbulent kinetic energy *k* and the turbulent dissipation rate *ε* are introduced to describe the turbulence behavior. The turbulent viscosity is modeled as follows:

1 $$\mu_{T}=\rho C_{\mu }\frac{k^{2}}{\varepsilon }\text{.}$$

The transport equations for *k* and ε are as follows:

2 $$\rho \frac{\partial k}{\partial t}+\rho u\cdot \nabla k=\nabla \cdot \left(\left(\mu +\frac{\mu_{T}}{\sigma_{k}}\right)\nabla k\right)+P_{k}-\rho \varepsilon \text{,}$$

3 $$P_{k}=\mu_{T}\left(\nabla u:\left(\nabla u+{\left(\nabla u\right)}^{T}\right)-\frac{2}{3}{\left(\nabla \cdot u\right)}^{2}\right)-\frac{2}{3}\rho k\nabla \cdot u\text{,}$$

4 $$\rho \frac{\partial \varepsilon }{\partial t}+\rho u\cdot \nabla \varepsilon =\nabla \cdot \left(\left(\mu +\frac{\mu_{T}}{\sigma_{\varepsilon }}\right)\nabla \varepsilon \right)+C_{\varepsilon 1}\frac{\varepsilon }{k}P_{k}-C_{\varepsilon 2}\rho \frac{\varepsilon^{2}}{k}\text{,}$$

with constants $C_{\mu }=0.09$, $C_{\varepsilon 1}=1.44$, $C_{\varepsilon 2}=1.92$, $\sigma_{k}=1.0$, $\sigma_{\varepsilon }=1.3$, where u is the velocity vector, and $\rho =998\;kg/m^{3}$ and dynamic viscosity μ= 1.0016 mPa s of water at 20^o^C.

The used model assumes Newtonian-type fluid, meaning that the viscous stress is directly proportional, with the dynamic viscosity as the constant of proportionality, to the shear rate. Density can vary concerning pressure, although it is assumed that the fluid is only weakly compressible, meaning that the Mach number is less than about 0.3. The *k-ε* equations are derived considering the flow has a high enough Reynolds number (critical *Re* = 2000). If this assumption is not fulfilled, both *k* and *ε* have very small magnitudes and behave chaotically so that the relative values of k and ε can change by large amounts due to small changes in the flow field. The *k-ε* model has historically been popular for industrial applications due to its reasonable convergence rate and relatively low memory requirements.

A number that expresses cavitation probability is the Cavitation number:

5 $$C_{A}=\frac{p-p_{v}}{\frac{1}{2}\rho v^{2}}\text{,}$$

where ρ is the density of the fluid, p is the local pressure, $p_{v}$ is the vapor pressure of the liquid v is the characteristic velocity of the flow. The value $C_{A}\ll 1$ means a significant probability of cavitation. The lowest value obtained here is 0.3, and a value below 0.8 is enough to observe cavitation [20].

**Fig. 1:**
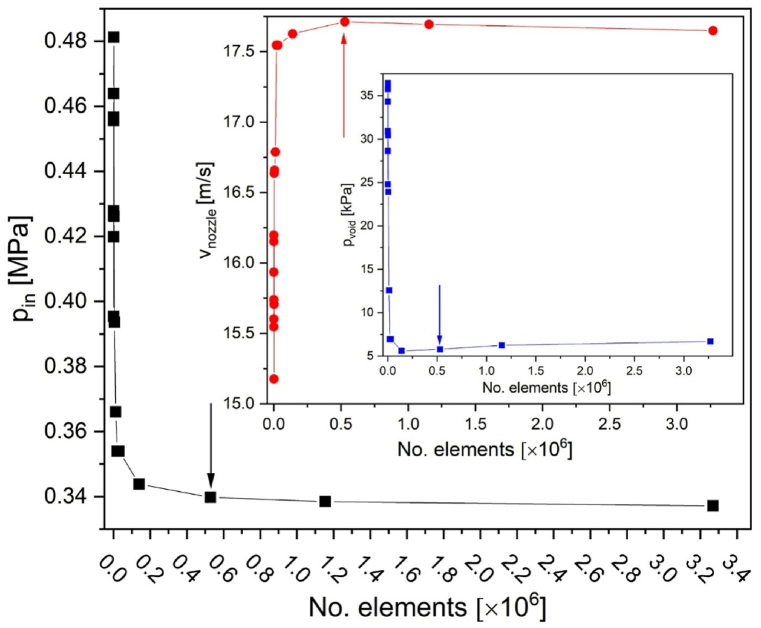
Selected pressures p_in_, p_void_ and velocity at the nozzle v_nozzle_ vs. no. of mesh elements. The arrow indicates the used mesh with 528345 elements.

**Fig. 2:**
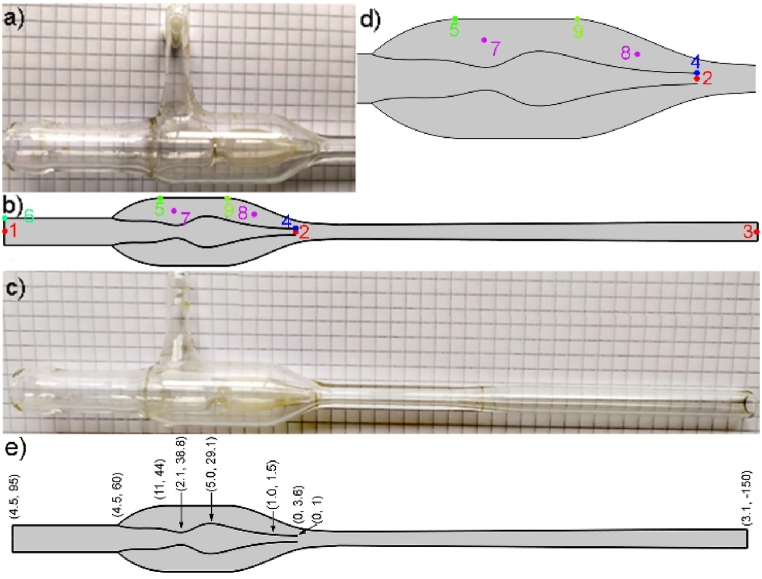
a) and c) photos of laboratory glass type water aspirator; b) and d) model with selected measurement points of pressure and velocity – 1, 2, 3 - points lying on the symmetry axis; 4 - edge of the nozzle, 5 and 9 - side of the aspirator, top and bottom respectively, 6 - side wall at entrance, 7 and 8 – inside of the side void, top and bottom respectively; e) model with marked coordinates (r, z) of some characteristic points; where r is the distance from symmetry axis in mm, and z is the height in mm, where nozzle's center is at coordinates (0, 0).

Due to axial symmetry only, half part of the cross-section was designed, and a 3D model was created by the 360° rotation around the vertical axis. The dimensions of the inlet (Φ = 9 mm), outlet (Φ = 6.2 mm), nozzle position (0, 0) and its nozzle diameter (Φ = 2 mm), converging angle (0.4^°^), diverging angle (0.5^°^) diameter of the tube around the nozzle at its height - throat (Φ = 7.2 mm) and total length (245 mm) together with some additional characteristic geometrical point coordinates is shown in Fig. 2e. Nozzle angles and lengths were taken with the caliper as faithfully as possible to reproduce the original device. The model consisted of 528345 triangle mesh elements with average and minimum element quality of 0.96 and 0.54, respectively (quality measure is skewness). Fig. 1 shows validation of the minimum number of mesh elements, after which results are independent of their number.

Further settings were: wall conditions – slip manual, turbulence intensity medium I_T_ 0.05, turbulence length scale L_T_ - geometry based with physically controlled tetrahedral mesh calibrated for fluid dynamic – extra fine. Two parameters were adjusted for the perfect fit of the experiment and model: slip velocity (−0.335 m/s, vertical direction *z-axis*) and outlet static pressure (100300 Pa). Static outer pressure is the laboratory atmospheric pressure and the p_4_ pressure for no-flow conditions (v_in_ = 0 m/s). The reference pressure was set to 0 Pa. The above-mentioned adjustable parameters are geometry-dependent. Slip condition modifies the fluid velocity at the wall. This velocity was used as a fitting parameter to match the measured pressures for maximal flow no-flow conditions in point 5 (Fig. 2b–d).

The experimental values measured are as follows: the maximum water velocity accessible was measured with a 2.5 L measuring cup, which was filled in ca. 25 sec. (repeated 10 times and averaged). Experimentally observed flows were in the 0.1–0.022 L/s range, corresponding to ca. 0.1–0.022 kg/s (ρ = 0.9983 kg/L at 20^o^C). The pressure was measured with chemistry pumping unit PC 3001 VARIO^pro^ EK Peltronic in the 100300 - 5740 Pa (±5 % error).

## 3 Results and discussion

The laboratory glass aspirator was measured and transferred to the model in the COMSOL program (Fig. 2a–c). The symmetry axis allowed the design in 2D (without the side arm, Fig. 2b–d, e). Additionally, velocity and pressure probe points depicted later in tables are marked by various colors in Fig. 2b–d. The nozzle geometry and water velocity are essential for the pressure drop [21]. Depending on the nozzle geometry, an additional cavitation effect (formation of gas bubbles) can be observed [22].

Table 1 describes probe points of pressure and velocity at specific points of the model; the results are presented in Table 2 for two cases with and without slip conditions. In general, the most straightforward flow profile without slip conditions on the wall is described by the Hagen–Poiseuille equation, which would show a parabolic velocity profile with maximum velocity in the center of the tube and due to shear stress and wall surface roughness lowers radially from the central axis reaching 0 at the wall. A slip condition at the wall was added to match the experimental results, increasing the liquid velocity at the wall by 0.335 m/s. The z-axis points to the top of the device, and water flows in the opposite direction. This condition flattens the parabolic velocity profile and allows the experimental data to be matched.

**Table 1:** The description of symbols used in text and tables.

1	v_in_ [kg/s]	The mass flow rate of water (point 1, Fig. 2b)
2	Slip [m/s]	Slip condition at the walls (max. value reaches −0.335 m/s)
3	p_1_ [Pa]	The pressure of water at the entrance – the point at the symmetry axis (point 1, Fig. 2b)
4	v_1_ [m/s]	Velocity magnitude at the entrance – the point at the symmetry axis (point 1, Fig. 2b–d)
5	p_2_ [Pa]	Pressure at the nozzle – the point at the symmetry axis (point 2, Fig. 2b–d)
6	v_2_ [m/s]	Velocity magnitude at the nozzle – the point at the symmetry axis (point 2, Fig. 2b–d)
7	p_3_ [Pa]	Pressure at exit – point at symmetry axis (point 3, Fig. 2b)
8	v_3_ [m/s]	Velocity magnitude at exit – point at symmetry axis (point 3, Fig. 2b)
9	p_4_ [Pa]	Pressure at the side's top – experimentally measured pressure (point 5, Fig. 2b–d)
10	v_4_ [m/s]	Velocity magnitude at the side's top (point 5, Fig. 2b–d)

**Table 2:** Pressure and velocity magnitudes in selected points of the model; the description in Fig. 2b–d.

1	2	3	4	5	6	7	8	9	10
v_in_ [m/s]	Slip [m/s]	p_1_ [Pa]	v_1_ [m/s]	p_2_ [Pa]	v_2_ [m/s]	p_3_ [Pa]	v_3_ [m/s]	p_4_ [Pa]	v_4_ [m/s]
Slip velocity −0.335 m/s									
0.10	−0.335	339769	1.57	14916	25.56	100290	2.95	5787	0.335
0.09	−0.335	283682	1.42	35170	22.36	100292	2.62	28116	0.335
0.08	−0.335	235062	1.26	52680	19.16	100294	2.28	47439	0.335
0.07	−0.335	193909	1.10	67448	15.96	100296	1.95	63757	0.335
0.06	−0.335	160215	0.94	79469	12.76	100297	1.62	77064	0.335
0.05	−0.335	133982	0.79	88748	9.55	100299	1.29	87367	0.335
0.04	−0.335	115229	0.63	95300	6.35	100299	0.96	94671	0.335
0.03	−0.335	103979	0.47	99148	3.14	100300	0.62	98986	0.335
0.02	−0.335	100205	0.31	100257	0.30	100300	0.29	100288	0.335
**Slip velocity 0 m/s**									
0.10	0	481968	1.57	−29595	32.06	100283	3.32	−46007	0
0.09	0	409516	1.42	−4888	28.85	100286	2.99	−18196	0
0.08	0	344680	1.26	17212	25.65	100289	2.65	6684	0
0.07	0	287465	1.10	36708	22.44	100292	2.32	28635	0
0.06	0	237877	0.94	53606	19.24	100294	1.99	47662	0
0.05	0	195914	0.79	67906	16.04	100296	1.66	63764	0
0.04	0	161569	0.63	79601	12.83	100297	1.33	76935	0
0.03	0	134842	0.47	88693	9.63	100298	1.00	87178	0

Table 2 shows data obtained for two cases with and without slip conditions. The experimentally measured pressure at the tenon corresponds to pressure on the top of the side wall p_4_ (Table 2, column 9, Fig. 2d - points 5). Points 8 and 9 in the side chamber (Fig. 2d) show identical values. The entire side/void region down to the nozzle level shows the same pressure accessible from outside. Without slip conditions (bottom part of Table 2), the pressure change is −46007 to 87178 Pa. After adjusting the outer pressure, it is possible to shift the values into the range 5787 (for v_in_ = 0.1 kg/s) – 100288 Pa (for 0.02 kg/s). The higher pressure for small flows should approach the atmospheric pressure of 100300 Pa (measured in the laboratory) and not cross it. The additional condition of wall slip allows it to fit into the experimentally achieved values of 5787–100288 Pa.

Table 3 shows the transition between values obtained in Table 2 for various slip conditions (top and bottom) for a single high flow rate of 0.1 kg/s.

**Table 3:** Pressures and velocity magnitudes of selected points for various mass flow rate v_*in*_ = 0.1 kg/s and wall slip velocities (Slip).

1	2	3	4	5	6	7	8	9	10
v_in_ [m/s]	Slip [m/s]	p_1_ [Pa]	v_1_ [m/s]	p_2_ [Pa]	v_2_ [m/s]	p_3_ [Pa]	v_3_ [m/s]	p_4_ [Pa]	v_4_ [m/s]
0.10	−0.34	339769	1.57	14916	25.56	100290	2.95	5787	0.33
0.10	−0.30	352564	1.57	10235	26.24	100289	2.99	665	0.30
0.10	−0.25	371636	1.57	3576	27.20	100288	3.04	−6677	0.25
0.10	−0.20	393433	1.57	−1559	28.18	100287	3.10	−13420	0.20
0.10	−0.15	414912	1.57	−7862	29.15	100286	3.15	−20978	0.15
0.10	−0.10	436661	1.57	−14780	30.12	100285	3.21	−28998	0.10
0.10	−0.05	458996	1.57	−22038	31.09	100284	3.26	−37348	0.05
0.10	0.00	481967	1.57	−29596	32.06	100283	3.32	−46007	0.00

The lowest measured pressure value is 5.7 kPa. It is above the theoretical vapor pressure of 2.3 kPa (at 20^o^C). Higher pressure measured can result from leaks and measurement error (±5 %). For the highest water velocity, the lowest pressure was obtained, and for no-flow conditions, side pressure (p_4_) should reach the measured atmospheric pressure of 100300 Pa. Minimal achievable pressure was limited by the maximal water network pressure achievable in the laboratory.

Cross-sections of pressure and velocity magnitude results are shown in Fig. 4, Fig. 5 along the aspirator's external wall (Fig. 4, inset - red line), symmetry axis (Fig. 5, inset – blue line), and internal wall (Fig. 6, inset – green line). Insets in the figures represent half of the aspirator model. Fig. 4 describes the changes in pressure (p_1_) and velocity magnitude (v_1_) at the external wall (marked red in the inset). In the initial part, pressure *p* and velocity *v* are constant. In general, pressure and velocity increase with the increase of mass flow. However, due to wall slip conditions, the velocity at the wall increases by 0.335 m/s, and pressure is slightly reduced. The velocity at the wall at the height of the nozzle drastically increases due to the presence of the central stream. The velocity gradient in the region from −12 to −27 mm (below the nozzle) is $0.29\frac{m}{s\;}/mm$ reaching maximum velocity ca. $4.3\frac{m}{s}$ at the wall. In contrast, the central stream velocity is ca. $25.6\;\frac{m}{s}$. The most critical side part shows pressure ranging from atmospheric for the no-flow condition to ca. 5.7 kPa for 0.1 kg/s in the entire side volume. After passing the nozzle, the pressure returns slowly to atmospheric pressure, and sudden velocity changes diminish in the next 50 mm.

**Fig. 3:**
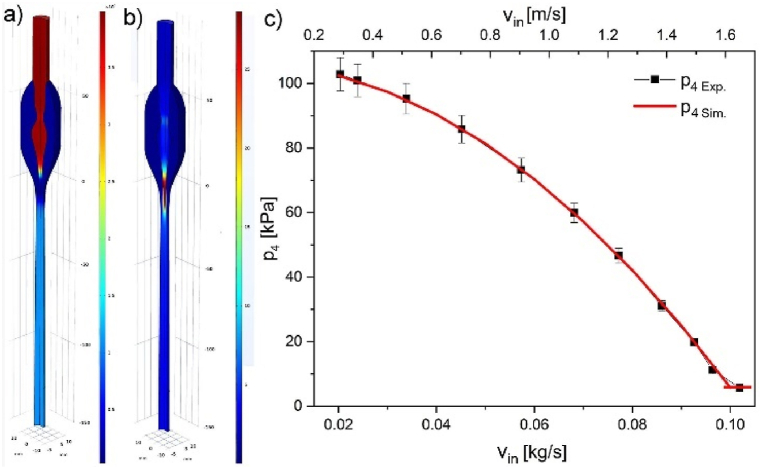
a) 3D COMSOL model with overlaid maps of a) pressure b) for water inflow of 0.1 kg/s; c) experimental values and simulation of pressure drop (20^o^C) vs. mass flow rate bottom axis is in kg/s and top axis is in m/s (from Fig. 2b–d, red point 5 – p_4_).

**Fig. 4:**
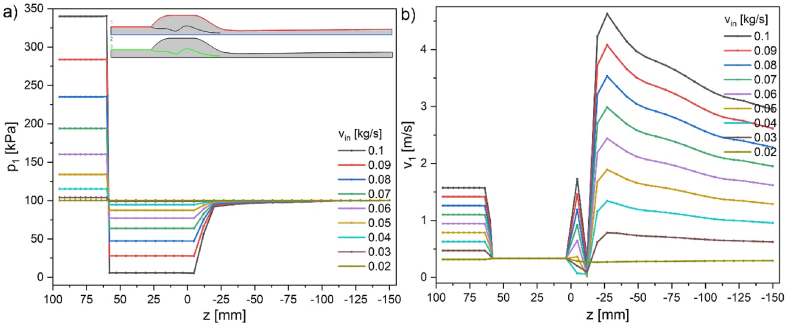
a) Pressure and b) velocity magnitude along the external wall for various inflow velocities in kg/s; cross-section 1-red in the inset.

**Fig. 5:**
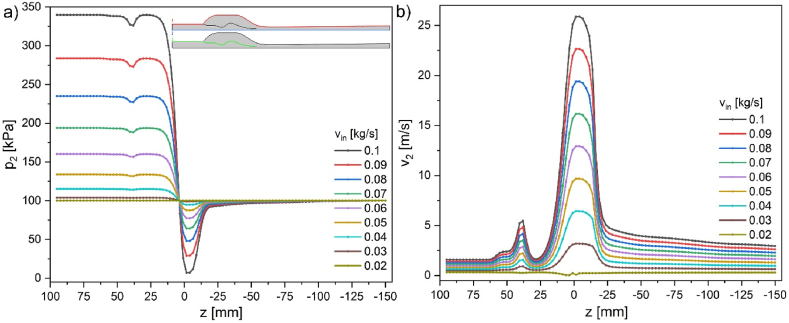
a) Pressure and b) velocity magnitude along the symmetry axis for various inflow velocities in kg/s; inset 2-blue cross-section.

**Fig. 6:**
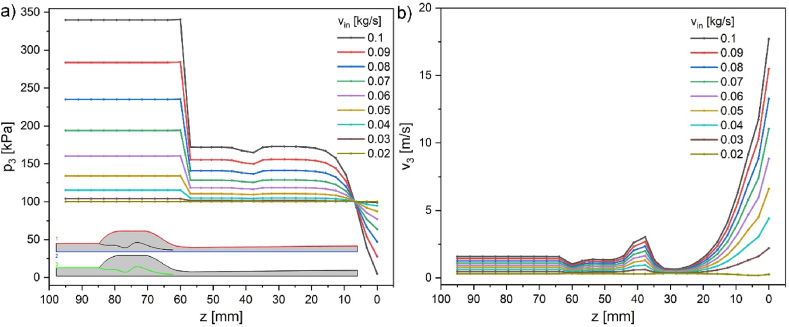
a) Pressure and b) velocity magnitude along the internal wall for various inflow velocities in kg/s; inset cross-section 3-green.

The average inflow velocity is ca. 1.58 m/s. Velocity increases with the decrease of flow diameter according to the energy conservation described by Bernoulli's equation mentioned in the introduction. The changes occur more intensely with an increased flow rate. The most significant increase in velocity magnitude appears in the narrowest part of the nozzle, reaching a maximum value of 25.6 m/s (Fig. 5b). The central part of this velocity (ca. 80 %) is reduced within the following 25 mm distance from the nozzle. Further decrease is much slower till the end of the aspirator. The nozzle region (0 to −25 mm) is the space where the influence of the under pressure from the side void and pressurized fluid stream interact, causing a decrease in the pressure on the symmetry axis (Fig. 5a).

The inside wall cross-section (Fig. 6) shows expected pressure and velocity changes dependent on the flow diameter. The zone where the pressure decreases and velocity increases reaches 25 mm above to −25 mm below the nozzle.

**Fig. 7:**
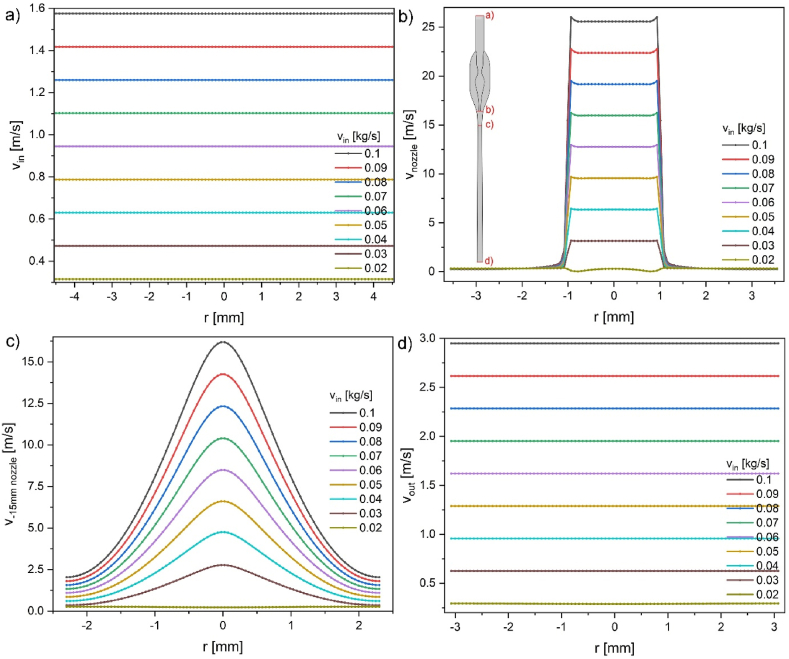
Velocity magnitude taken at selected cross-sections perpendicular to the long axis of the aspirator: a) entry (z = 95 mm); b) nozzle (z = 0 mm); c) at max. velocity (z = −15 mm); d) exit (z = 150 mm). The inset in b) shows the marked positions of the cross-sections.

Perpendicular cross sections of velocity profiles show how the velocity magnitude looks across the channel length (Fig. 7). Four specific positions were selected for comparison: a) entry, b) nozzle, c) −15 mm, which is below the nozzle (max. velocity magnitude), d) at the exit. All the cross-sections are marked by red lines on the model in the inset in Fig. 7b.

Flattened velocity profiles are due to additional slip conditions on the walls. Lower inflow velocity than at the exit is due to the difference in tube diameter, which is 9 and 6.2 mm, respectively (Fig. 7a–d). The velocity profile at the nozzle (Fig. 7b) shows the maximum velocity value at the nozzle output, which is ca. 25.6 m/s. This velocity tends to decrease to zero outside the nozzle (Fig. 7b). The cavitation number $C_{A}$ is 0.3 for this nozzle geometry and the maximum flow velocity. This value can be roughly estimated by the p_2_ pressure ratio at the outlet to inlet 100.3/340≈0.3 (Fig. 5a) [20]. Parameters increasing the cavitation effect are a smaller outlet angle, large diameter ratio of the throat to the nozzle, large throat velocity, and more dissolved gas in liquid [22]. Fig. 7b shows the interface between liquid and air phases. Experimentally, this side/void region is filled with low-pressure air. Water gets into this void only when the water flow stops abruptly. Due to a vacuum in the side part, the remaining water is redirected to the tenon. The simplified model used here indicates a lack of fluid movement (Table 2) velocities v_7_ and v_8_ for no-slip conditions and 0.335 m/s with slip conditions set as bias value on the wall (although no water is present in this void). Fig. 7c shows the velocity profile 15 mm below the nozzle. In this section, the velocity magnitude is still high in the central tube part (nozzle diameter is 2 mm), the outer tube diameter is 5.0 mm, and it broadens up to 6.2 mm at the device exit. The velocity profile is still strongly influenced by the nozzle geometry. The velocity field returns to its flattened profile during the next 140 mm until exit (Fig. 7d).

**Fig. 8:**
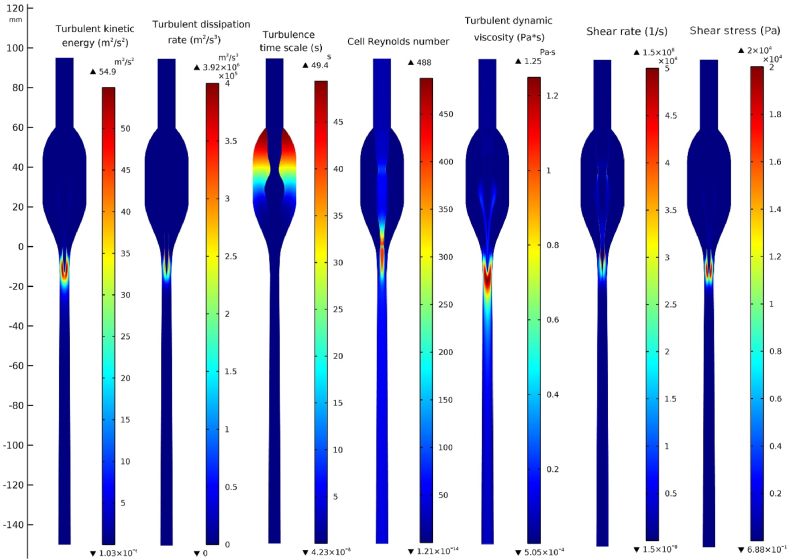
Multiple turbulent model parameters characterizing the system for inflow velocity v_in_ 0.1 kg/s and slip conditions −0.335 m/s. Description in the image. The scale is placed right from the map with visible maximum and minimum values.

**Fig. 9:**
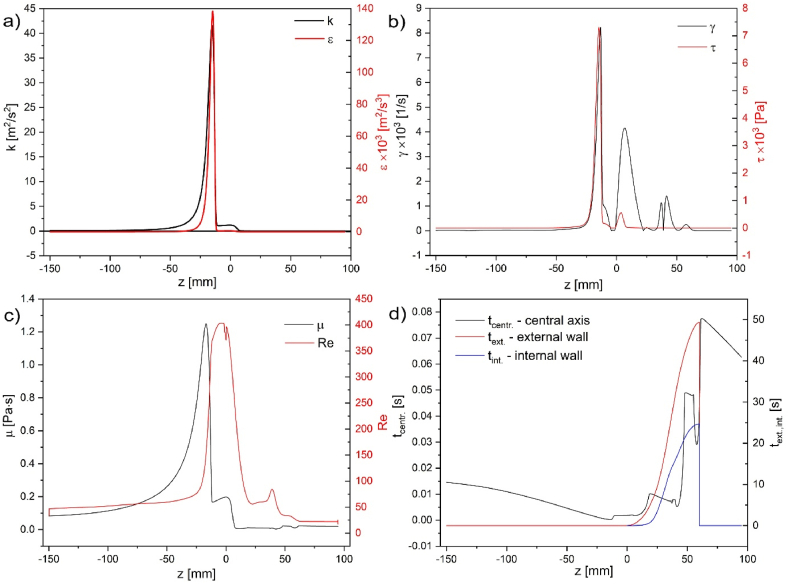
Linear plots of turbulent model parameters along the central symmetry axis.

Fig. 8 indicates the main parameters describing turbulence in the selected model. Most of them reach the maximum just after fluid leaves the nozzle, e.g., turbulent kinetic energy (*k* = 54.9 m^2^/s^2^) is the mean kinetic energy per unit mass associated with fluid swirling - eddies, its dissipation rate (ε = 3.92 · 10^6^ m^2^/s^3^), Reynolds number (Re_max_ = 488), turbulent dynamic viscosity (μ = 1.25 Pa · s), shear rate (γ = 1.5 · 10^6^ 1/s), and shear stress (τ = 2 · 10^4^ Pa)(Fig. 8, Fig. 9).

The maximum value of shear rate (γ) and shear stress (τ=μ·γ) lie on the nozzle's outside wall and not on the central axis presented in Fig. 9b. Only the turbulence time scale reaches its maximum in the side void ($t_{\max.}$ = 49.4 s, and $t_{\min.}$ = 4.2 · 10^−6^s). The minimal turbulence time scale corresponds to the region with maximal shear strength non-central and below the nozzle. For maximal *Re* numbers obtained in the system, a developed turbulent flow does not appear (Re_crit._ = 2300) [47].

Fig. 9 presents quantities in Fig. 8 lying on the central symmetry axis. The values are different due to the nature of the quantities. Not all of them reach the maximum on the central axis.

With the rise of water temperature, the *Re* number also increases for v_in_ = 0.1 kg/s (for 274 K), its maximal value is 280 (293.15 K, 488), and for 370K, it reaches 1640. Using the following formula for turbulence intensity derived from an empirical correlation for pipe flows $I=0.16*{Re}^{-\frac{1}{8}}$ [48] for the highest *Re* number the intensity reaches 6.3 % where turbulence intensity of 1 % or less is generally considered low and above 10 % as high [48].

**Fig. 10:**
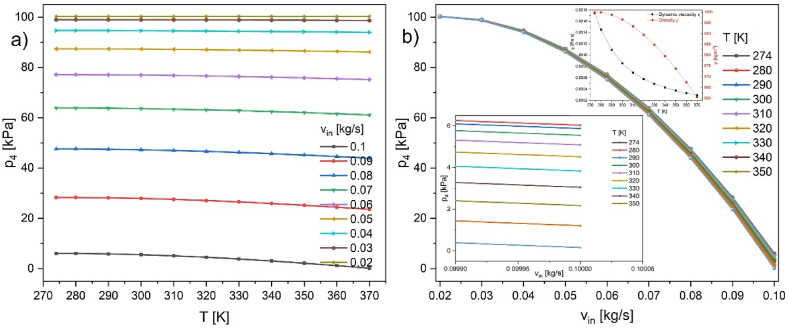
Dependence of the pressure drop on the side of the pump (point 5 Fig. 2) for a) various inflow velocities in kg/s and b) temperature in the range 274–370 K (0.85–96.85^o^C); inset temperature dependences of water's density and dynamic viscosity. Temperature dependences are described as follows: Density ρ [kg/m^3^] T (273.15–293.15K); y = 0.00006ּ T^3^ - 0.060 ּ T^2^ + 18.92 ּ T - 950.70; T (293.15–373.15 K); y = 0.000010 ּT^3^ - 0.013ּ T^2^ + 4.97ּ T + 432.26; Dynamic viscosity η [Paּs ] T (273.15–413.15K); y = 1.38 - 0.021ּ T + 1.36 ּ10^−4^ּ T^2^ - 4.65 ּ10^−7^ּ T^3^ + 8.90 ּ10^- 10^ּ T^4^ - 9.08 ּ10^−13^ּ T^5^ + 3.85 ּ10^−16^ּ T^6^; T (413.15–553.75K); y = 0.0040–2.11 ּ10^−5^ּ T + 3.86 ּ10^−8^ּ T^2^ - 2.40 ּ10^−11^ּ T.^3^.

Additionally, the influence of temperature rise on the achievable pressures was studied (Fig. 10a and b, and Table 4, Table 5). The model assumes changes (decrease) in water density and dynamic viscosity according to the inset plot in Fig. 10b. Depicted is the temperature dependence of pressure on the side wall characteristic point “5” marked as p_4_ (Fig. 2b–d, Table 2) in the temperature range 274–370 K. The obtained p_4_ pressures are 6132 - 158 Pa in the respective temperature range. The relative change of the final pressure is of the order (6132-158)/6132 = 0.974. From a theoretical point of view, an increase in water temperature can decrease the p_4_ pressure. It could seem an improvement, reaching 158 Pa. Still, the limiting factor is the water vapor pressure mentioned in the introduction, which also increases with temperature (for 90^o^C, it is 70 kPa, and for 100^o^C, it is 101.3 kPa [40]), nullifying potential gain. This is the second parameter the program does not consider automatically.

**Table 4:** Temperature dependence of p_4_ pressure for various inflows (slip wall condition - 0.335 m/s).

T [K]	Mass flow rate [kg/s]								
	0.1 [kg/s]	0.09 [kg/s]	0.08 [kg/s]	0.07 [kg/s]	0.06 [kg/s]	0.05 [kg/s]	0.04 [kg/s]	0.03 [kg/s]	0.02 [kg/s]
	p_4_ [Pa]								
274	6032	28309	47590	63870	77145	87420	94701	98998	100287
280	6034	28311	47592	63871	77146	87421	94701	98998	100287
290	5875	28183	47493	63797	77093	87386	94682	98990	100287
300	5548	27922	47289	63644	76985	87316	94642	98974	100288
310	5089	27553	47002	63430	76833	87216	94585	98950	100289
320	4515	27093	46644	63162	76642	87092	94515	98922	100291
330	3834	26547	46219	62843	76416	86944	94431	98887	100292
340	3052	25920	45731	62477	76157	86774	94334	98847	100294
350	2174	25216	45183	62067	75865	86583	94225	98801	100296
360	1208	24440	44579	61614	75543	86372	94105	98751	100297
370	158	23599	43923	61121	75194	86143	93973	98696	100299

**Table 5:** p_4_ pressure vs. inflow rates for various temperatures (slip wall condition - 0.335 m/s).

v_in_ [kg/s]	Temperature [K]										
	274 [K]	280 [K]	290 [K]	300 [K]	310 [K]	320 [K]	330 [K]	340 [K]	350 [K]	360 [K]	370 [K]
	p_4_ [Pa]										
0.1	6032	6034	5875	5548	5089	4515	3834	3052	2174	1208	158
0.09	28309	28311	28183	27922	27553	27093	26547	25920	25216	24440	23599
0.08	47590	47592	47493	47289	47002	46644	46219	45731	45183	44579	43923
0.07	63870	63871	63797	63644	63430	63162	62843	62477	62067	61614	61121
0.06	77145	77146	77093	76985	76833	76642	76416	76157	75865	75543	75194
0.05	87420	87421	87386	87316	87216	87092	86944	86774	86583	86372	86143
0.04	94701	94701	94682	94642	94585	94515	94431	94334	94225	94105	93973
0.03	98998	98998	98990	98974	98950	98922	98887	98847	98801	98751	98696
0.02	100287	100287	100287	100288	100289	100291	100292	100294	100296	100297	100299

Table 4, Table 5 contain data in Fig. 10. These data show the applicability limit of the *k-ε* model inserted in COMSOL.

## 4 Conclusions

Computational fluid dynamic modeling (CFD) can provide additional insight into the system, which can only be measured directly by influencing the performance of the equipment and, of course, additional financial expenditure. It was possible to describe the Venturi effect well using the *Turbulent flow k-ε* model. The Venturi effect shows itself as a decrease of pressure in the side void volume where the tenon in laboratory equipment is located. As expected, for larger water flow velocities (or volume), a more significant p_4_ pressure drop is shown. The experimentally measured pressure drops on the tenon for various water flows led to adjusting model parameters and reaching the ideal fit of the experimental data (Fig. 3c). The parameters adjusted were slip velocity (−0.335 m/s), outlet static pressure (100300 Pa), and reference pressure set to 0 Pa. This calibration allowed the generation of realistic pressure, velocity, and turbulence parameters maps, showing the benefits of CFD modeling.

In the temperature-dependent study, results show a potential decrease in tenon pressure due to the decrease of dynamic viscosity and water density with temperature increase. The program does not consider the increased vapor pressure of water with increased temperature, which nullifies this potential benefit and shows limits to the model.

The lack of water in the side void shows itself in the model by zero water velocity and the maximal turbulence time scale. Apparent fluid velocity on the wall in the side void is caused only by imposed slip conditions on all walls.

The change of pressure at the nozzle from atmospheric to ca. 5.7 kPa is enough to imply cavitation conditions ($C_{A}\approx 0.3$) and fluid mixing.

At the boundary-nozzle tip and side void (interface water/air), a vast velocity difference is observed of ca. 25.6 $\frac{m}{s}$ (Fig. 7b). The nozzle geometry is a significant perturbation in the velocity and pressure field, which maximum can be found in the range 25 mm to −25 mm on the z-axis. It is also the region where most turbulence parameters reach their maximum Fig. 8.

## CRediT authorship contribution statement

**Krzysztof Tadyszak:** Writing – original draft, Validation, Formal analysis, Data curation, Conceptualization. **Mariia Rabyk:** Writing – review & editing, Methodology, Investigation, Formal analysis. **Jiří Pánek:** Writing – review & editing, Writing – original draft, Supervision. **Martin Hrubý:** Writing – review & editing, Writing – original draft, Supervision, Methodology.

## Data availability statement

No new data were created or analyzed in this study. Data sharing does not apply to this article.

## Declaration of competing interest

The authors declare the following financial interests/personal relationships which may be considered as potential competing interests: Jiri Panek reports financial support was provided by Ministry of Education Youth and Sports of the Czech Republic. Martin Hruby reports financial support was provided by Ministry of Education Youth and Sports of the Czech Republic. If there are other authors, they declare that they have no known competing financial interests or personal relationships that could have appeared to influence the work reported in this paper.
